# Correction to: Influences on COVID-19 booster uptake among adults intending to receive a booster: a qualitative study

**DOI:** 10.1093/heapro/daag113

**Published:** 2026-07-27

This is a correction to: Ramey Moore, Rachel S Purvis, Don E Willis, Ji Li, James P Selig, Jeanne Ross, Pearl A McElfish, Influences on COVID-19 booster uptake among adults intending to receive a booster: a qualitative study, *Health Promotion International*, Volume 39, Issue 3, June 2024, daae067, https://doi.org/10.1093/heapro/daae067

The funding statement for this paper lists the grant number: “10T2HL156812-01” for the Community Engagement Alliance (CEAL) funding acknowledgment. This should read: “OT2HL158287”.

The error has been outlined only in this correction notice to preserve the version of record.

